# *CatMass*: software for calculating optimal sample masses for X-ray absorption spectroscopy experiments involving complex sample compositions

**DOI:** 10.1107/S160057752300615X

**Published:** 2023-08-18

**Authors:** Jorge E. Perez-Aguilar, Ash Caine, Simon R. Bare, Adam S. Hoffman

**Affiliations:** a Stanford Synchrotron Radiation Lightsource, SLAC National Accelerator Laboratory, 2575 Sand Hill Road, Menlo Park, CA 94025, USA; University of Essex, United Kingdom

**Keywords:** *CatMass*, XAS, catalysts, sample preparation optimization

## Abstract

This paper presents software for calculating the optimal mass of samples with complex compositions (*e.g.* supported metal catalysts) for X-ray absorption spectroscopy (XAS) and scattering measurements. The ability to calculate the sample mass and other relevant parameters needed for an XAS measurement allows experimentalists to be better prepared in terms of detector selection, energy range of scan and overall time needed to complete the measurement, thus increasing efficiency. *CatMass* builds on existing sample mass calculators allowing users to determine the optimum sample preparation, collection geometry, usable energy range for a scan and approximate edge step of the absorption event. Visualization tools present the absorption calculation results in a format familiar to XAS experimentalists, with the added ability to save calculations and plots for future reference or recalculation. *CatMass* is a program broadly applicable in catalysis and is helpful for users with complex samples due to composition/stoichiometry or multiple competing elements.

## Abstract 2

*CatMass* is a new software for calculating the optimal sample mass of samples with complex compositions (*e.g.* a supported metal catalyst mixed with a diluent) for X-ray absorption and X-ray scattering experiments with supplemental results to guide experimental design and collection geometry.

## 1. Purpose/introduction

The quality of X-ray absorption spectroscopy (XAS) data is dependent on the uniformity (homogeneity), mass of the sample and the material surrounding the sample probed by the X-ray beam. This is especially true for transmission XAS experiments, where Beer’s law assumes all incident photons, that pass through an aperture that defines the beam size, interact with the same amount of material. In experimental application, this allowed the signal-to-noise ratio for transmission XAS to be determined with a maximum when the total absorption cross-section of the sample is approximately 2.6 above an absorbing edge (Iwasawa, 1986 ▸; Stern & Kim, 1981 ▸). More recent works present a range for the recommended total absorption cross-section from 1 to 2.5, but less than 3 (Calvin, 2013 ▸). The reduction in the optimal sample total absorption above the edge from 2.6 to as low as 1 is to allow for absorption from other materials in the beam path such as the windows on an *in situ* experimental cell. Determining the photoabsorption cross-section, and ultimately the desired absorption length, is easily achieved for samples consisting of a single element, as the photoabsorption cross-section can be quickly found using online resources (Henke *et al.*, 1993 ▸). From this value the sample mass is calculated knowing the photon energy, desired absorption length above the absorbing edge and the cross-section of the sample holder perpendicular to the incident beam. Aside from photon energy, other parameters are tunable, and a potential challenge, when preparing for XAS experiments.

Sample holders come in a variety of shapes and sizes and are normally custom made to optimize factors that include the needs of the experiment (*e.g.* flow geometry) and the sample volume optimized for the properties of the X-ray beam at the synchrotron (X-ray path length). *Ex situ* sample holders often allow the sample to be packed so that the sample thickness can be readily adjusted for the desired absorption length. In contrast, *in situ*/*operando* sample holders have fixed geometries often requiring a defined volume of material (Bare & Ressler, 2009 ▸; Clausen *et al.*, 1991 ▸). It is often the case that this may result in the sample holder containing either more or less than the idealized amount of sample and thus over- or under-absorb, resulting in a distortion of the X-ray spectra and/or a diminished signal-to-noise ratio. For over-absorbing samples it is common practice to either design experimental cells with variable volumes (Hoffman *et al.*, 2018 ▸; Chupas *et al.*, 2008 ▸) or dilute the sample with a less-absorbing (more X-ray transparent) material (*e.g.* BN, cellulose, SiO_2_). If the choice is made to dilute the sample, then this allows the absorption length to be tuned, and ultimately the mass of sample in the beam for a fixed sample geometry. However, dilution introduces additional challenges in determining the appropriate sample-to-diluent ratio and increases the complexity of calculating the diluted sample cross-section.

Determining the photoabsorption cross-section for multi-element samples is less straightforward compared with their single elemental counterparts. In the catalysis field, for example, catalyst sample compositions often consist of an element of interest that is supported on a high surface area support [*e.g.* Pt nanoparticles supported on alumina (Aitbekova *et al.*, 2022 ▸)]. This may then be additionally diluted for optimal kinetics measurements or packing in a fixed geometry cell during an *operando* XAS experiment, for example, TiO_2_-supported Co particles diluted with mesoporous silica (SiO_2_) (Van Ravenhorst *et al.*, 2021 ▸) or carbon-supported FeNi particles (Acharya *et al.*, 2022 ▸). To determine the sample mass, it is common practice to use a weight-averaged photoabsorption cross-section assuming that the system is homogeneous in composition [equation (1)], where μ_ave_(*E*) is the average photoabsorption cross-section, and *x_i_* and μ_i_(*E*) are the mass fraction and photoabsorption cross-section of element *i* in the sample, respectively,

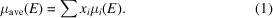

Though this assumption is reasonable, it is up to the experimenter to practice good sample preparation techniques to ensure the sample, and potential diluent, are as homogeneously mixed as possible.

The challenge in calculating the optimal sample mass required for a transmission experiment poses an opportunity for software to aid in these calculations. *Hephaestus*, part of the *Demeter* package (Ravel & Newville, 2005 ▸), and *XAFSMass* (Klementiev & Chernikov, 2016 ▸) are examples of software made available to users for determining the absorption properties of stoichiometric compounds and samples with complex compositions. Although broadly applicable to the XAS user community, these packages lack some functionality required by the catalysis community such as (i) accounting for diluents and (ii) identifying how elements of similar atomic number in the sample or other edges of an element may influence the usable photon energy range employed for the XAS measurement.

Herein we present *CatMass*, a software tool to aid catalyst and materials experimentalists in determining the required sample mass for XAS and X-ray scattering experiments. The overall purpose of the tool is to readily calculate the optimum mass of material that is needed for the desired experiment, to guide the user when deciding if the measurement should be transmission or fluorescence based, and to identify the usable scan range by highlighting competing edges that can be attributed to other elements in the sample or other edges of the absorber. This software has expanded capabilities compared with previously reported software by allowing for more complex sample composition inputs and providing graphical feedback to guide sample preparation. This software has been heavily used by the Consortium for Operando and Advanced Catalyst Characterization via Electronic Spectroscopy and Structure (Co-ACCESS) (Bare & Hong, 2023 ▸) catalysis user community at the Stanford Synchrotron Radiation Lightsource (SSRL) as is highlighted in the examples below.

## 2. Parts of *CatMass*

### 2.1. Language, modules and machine requirements

*CatMass* is available in two formats for user installation. A Microsoft Windows executable can be downloaded through the Co-ACCESS website (Hoffman, 2023 ▸). The development version is written in Python 3.9.7 and can be obtained from GitHub (Hoffman, 2021 ▸). The graphical interface was built using *PyQt5* with *Numpy* and *Matplotlib* being utilized for visualizing the results. The modules *xraydb* (Newville *et al.*, 2023 ▸) and *xraylib* (Schoonjans *et al.*, 2011 ▸) are used for determining the bulk material properties, chemical formula parsing and elemental photoabsorption cross-section.

The Windows executable version of the software requires a PC running Microsoft Windows (currently verified on Windows 7, 10 and 11) and 0.5 Gb of available hard drive space for installation. The development version requires a Python 3.9.7 environment with appropriate modules installed.

### 2.2. GUI overview

**Figure 1:**
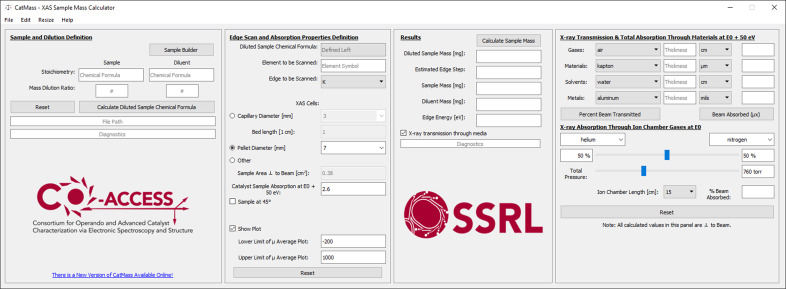
Main window of *CatMass* with the ‘X-ray transmission through media’ box checked, showing all four input and results panels. When the program starts, 2.6 is the default value for the for total absorption, and a 7 mm pellet as the XAS cell is selected. When an *in situ* capillary cell is selected, the default bed length is 1 cm. The default plot limits are −200 and 1000 above the edge energy of the element to be scanned. The ‘Diagnostics’ boxes are populated when an input is missing for the calculation the finish. Information is generally filled out in each panel top to bottom, then passed to the next panel on the right and so on. All information can be saved or reset/cleared under the ‘Save’ features in the ‘File’ or ‘View’ drop down menus, respectively. ‘Reset’ buttons will reset values to the local panels only. Fig. 4 ▸ provides an overview on how to progress through the calculator and obtain a result.

*CatMass* displays several windows that are used to find the optimal sample mass for an XAS measurement. The main window, shown in Fig. 1 ▸, contains four sections guiding the user from sample composition, X-ray measurement properties, calculated results and determining absorption from common beamline components during a measurement. The ‘Sample and Dilution Definition’ panel is intended for the user to input the chemical formula of a bulk, stoichiometric compound and a diluent if required using the dilution ratio input boxes.

**Figure 2:**
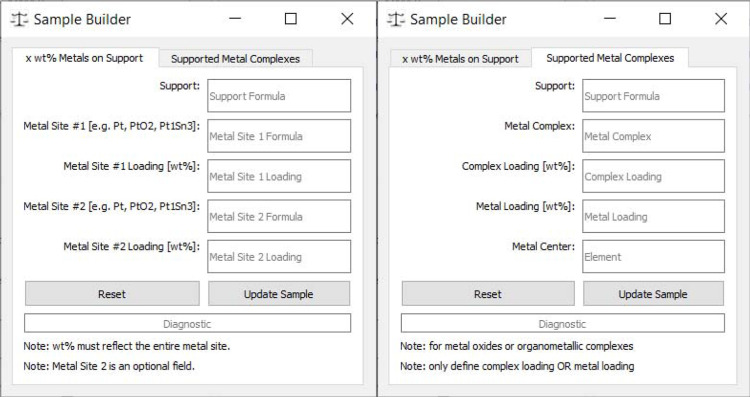
Sample builder view of *CatMass* for more complex samples. Each white text box is an input available to build the sample. Reset buttons will clear values to the local tab only. Examples of common inputs are described in Fig. S1 in the SI.

If the sample is more complex (*e.g.* 1 wt% Pt on Al_2_O_3_), then the ‘Sample Builder’ button opens a new window with additional inputs to build the complex sample (Fig. 2 ▸). The new window allows samples to be built based on defining the support and metal(s) or metal complex on their support based on their weight fraction. Examples of how to input various catalyst samples can be found in the supporting information (SI). When ‘Update Sample’ is selected in the ‘Sample Builder’ window, the information in this window is converted to a stochiometric formula and is passed to the main window.

**Figure 3:**
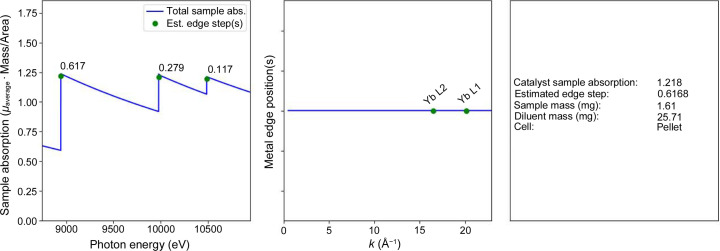
Visual results window of *CatMass*. The example is a pellet of Yb_2_O_3_ diluted with cellulose in a 1:16 ratio. The left panel displays the estimated edge steps of Yb_2_O_3_ at its absorption events. The middle panel displays where the competing absorption events are (Yb *L*­_II_- and *L*_I_-edges) in *k*-space. The right panel displays information from the calculator for sample preparation. The sample is optimized for transmission at 90° relative to the beam path at the *L*_III_- and *L*_II_-edges and is rotated at 45° relative to the beam path for fluorescence at the *L*_I_-edge. Details on the optimization are discussed in Section S2 of the SI.

The ‘Edge Scan and Absorption Properties Definition’ panel contains inputs for the specific photon energy used to calculate the sample mass, details about experimental cell/sample holder size and geometry with respect to the incident beam; the desired absorption length of the sample; and the plotting range for visualizing the absorption event. The energy of the calculation can be defined by the element–edge pair for XAS measurements, or through defining a specific energy for X-ray scattering measurements. Total sample absorption length is a tunable parameter that will be discussed further in the example below. A starting value for the total absorption length of 2.6 is recommended for transmission XAS experiments, assuming absorption from all other materials (reactor walls, air) is negligible around the energy range of interest. The sample area field has several common geometries for standard pellet diameters (5, 7, 10 and 13 mm) and capillary diameters (1, 2 and 3 mm) used for *in situ* flow systems for quick selection. These fields can also be edited if the user has a different diameter pellet or capillary. A custom sample area can also be defined if needed. Selecting the ‘Sample at 45°’ check box creates a slider that allows the user to rotate the sample from perpendicular to parallel to the beam, at a default of 45°, and calculates a projected area and total absorption cross-section given the angle, allowing for quick comparison of transmission versus fluorescence geometries. These projected areas are then used in the sample mass calculation. Checking the ‘Show Plot’ box at the bottom of the panel will generate a visual representation of the results in a new window (Fig. 3 ▸) when the calculation is run. A starting range from −200 to 1000 eV from the edge energy is typical for an XAS measurement but can be modified depending on the complexity of the sample with multiple absorption events. The plots generated are (i) the approximate total absorption across the energy range, identifying all additional adsorption events in the range and their approximate edge steps; and (ii) a *k*-space plot with the additional absorption events identified in *k*. The ‘Reset’ button at the bottom of the panel resets all the parameters to the default values present when opening the software.

The ‘Results’ panel is initially empty but, after a calculation is run, results pertaining to the sample and diluent mass, the estimated edge step of the absorbing element and the energy at which the calculation was performed are presented. The ‘Calculate Sample Mass’ button at the top of the panel runs the calculation given the inputs in the first two panels, returning results in the text fields below the button and in the plot window, if selected. All the values used to perform these calculations, as well as the results, can be saved as a text file and can be reimported for future modification into *CatMass*. Plots (shown in Fig. 3 ▸) can also be saved as images.

The last panel, which is only accessible when the ‘X-ray transmission through media’ box is checked, organizes common materials used for XAS experiments in several drop-down menus. Common gases (*e.g.* He, N_2_, Ar), materials [*e.g.* Kapton^®^, quartz, polyether ether ketone (PEEK)], solvents (*e.g.* water, acetone, hexane) and metals (*e.g.* Al, Pb, Be) can be selected. Based on the thickness input of the material of interest, the percentage of the beam transmitted, and the absorption, can be calculated at 50 eV above the energy specified in the absorption calculation input. This allows the user to calculate the absorption lengths of non-sample materials located between ion chambers, using the results to adjust (reduce) the total absorption of their sample if the experimental cell or local environment is a non-negligible absorption length. Additionally, the percentage transmission through ion chambers (of fixed or custom length) with different gas mixes and/or total pressures can also be calculated at the specified energy.

## 3. Using *CatMass* to determine how to prepare a sample and record an XAS scan

### 3.1. General XAS sample characteristic guidelines when using *CatMass* and any XAS sample

Performing a good-quality and efficient XAS experiment requires more planning than simply placing a sample in the X-ray beam and collecting a spectrum. Aside from determining the amount of sample to place in the beam path, considerations also must be made regarding collection geometry, transmission or fluorescence, as well as the necessary energy range that should be scanned. This has resulted in the XAS community establishing a few useful guidelines that will likely result in a successful, good-quality XAS measurement. When determining how to prepare a new powder sample, it is common to assume to attempt measurement as a transmission experiment if the absorbing atom of interest is greater than 1 wt% of the sample. As noted above, the optimal total absorption for a sample is 2.6 absorption lengths (μ*x* = 2.6), determined above the edge [approximate edge energy (*E*_0_) + 50 eV]. While this ensures the optimum amount of absorption by the sample, it does not determine whether the contribution from the absorption event of interest is strong enough to generate a quality XAS spectrum. At this point the edge step, determined by comparing the total absorption above and below the edge (at approximately *E*_0_ ± 50 eV), can be calculated. Quality transmission XAS spectra generally have an edge step of 0.2 to 1.0. The two constraints, a total absorption approximately equal to 2.6 with an edge step between 0.2 and 1, create an optimization problem as these two parameters are dependent on the composition of the entire sample. An added complication that is not resolved in the calculation of the total absorption or edge step is the approximation of the white line intensity. For some edges, notably the fifth-row transmission metal *L*-edges, the white line intensity may be substantially larger than the step in high oxidation states, *e.g.* the Re *L*_III_-edge of Re^7+^ (Qi *et al.*, 2020 ▸). Scanning over the energy region of this intense feature can result in very few photons transmitted through the sample, resulting in a poor quality or distorted signal that is not representative of the sample if the sample was prepared assuming a total absorption length of 2.6. Given the current inability to predict such events, literature surveys or a test experiment need to be performed to help guide a reasonable absorption length selection. If the sample cannot be optimized for transmission, or it appears to have a weak edge step compared with the total absorption, the measurement must be made with a fluorescence detector, possibly including a transmission measurement to detect for self-absorption (Trevorah *et al.*, 2019 ▸; Newville, 2014 ▸). Quality measurements also depend on the data point density collected during the pre-edge, edge (X-ray absorption near-edge structure) and post-edge (extended X-ray absorption fine structure) region of the sample (Calvin, 2013 ▸; Newville, 2014 ▸) and are outside the scope of this software and with the transition to continuous scan versus step scan is becoming less of a concern.

### 3.2. *CatMass* workflow

**Figure 4:**
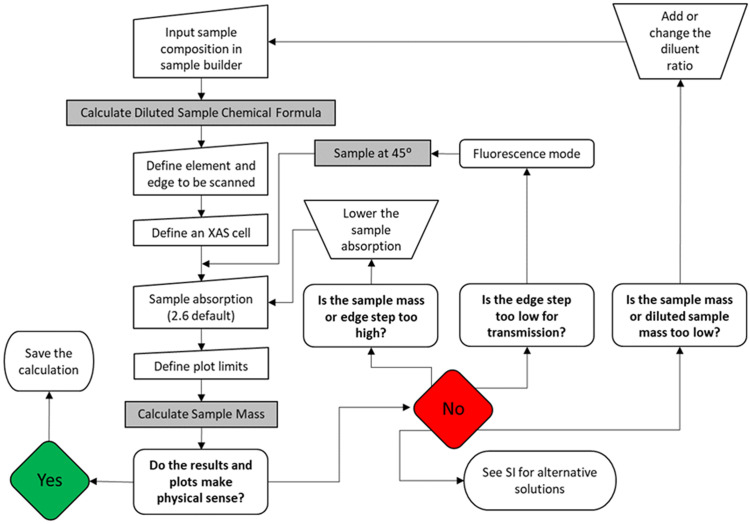
*CatMass* workflow to determine the optimal sample preparation for X-ray measurements. The grey boxes are the buttons to update the calculation. The questions in the workflow refer to the XAS cell to be used or the constraints given in Section 3.1. Alternative solutions are shown in Section S3 of the SI.

*CatMass* allows for the quick assessment of sample mass requirements and absorption and edge step determinations to guide the user in how to prepare and collect an XAS spectrum using the guidelines above. The workflow to determine the ideal sample and scan parameters is presented below (Fig. 4 ▸). This workflow starts by defining (i) the sample and its potential dilution, (ii) the X-ray energy range and (iii) the sample geometry (area and angle to beam) that will be used for the measurement; this returns an initial result. Based on these results the user can iterate through the inputs, recalculating the mass and edge step until a reasonable transmission sample can be prepared, or it is concluded that a fluorescence measurement is required. For more complex samples (samples containing multiple competing elements or edges), a more detailed assessment of sample absorption and edge steps can be visualized by plotting these calculations over a selected energy range using the plotting option. The iterative process is then continued until the sample meets the requirements listed in Section 3.1 for quality XAS spectra at all absorption edges scanned. Lastly, all the input information and results can be saved as a text file for future reference or modifications.

### 3.3. Example results from *CatMass* compared with XAS measurements

Table 1 ▸ shows four examples of the optimized packing and scan parameters determined from *CatMass* for a set of samples and reference compounds. These examples were selected to show the versatility of the software as well as a sampling of its use in the XAS user community at the Stanford Synchrotron Radiation Lightsource. Given the constraints of the sample composition or thickness, XAS sample holder and edges to be measured, several iterations through Fig. 4 ▸ were conducted to achieve the best sample preparation criteria. Selected examples from Table 1 ▸ are worked out using the *CatMass* software in the SI. The data for Yb_2_O_3_ in Table 1 ▸ show the power of *CatMass* to quickly identify a means for preparing a sample to allow collection on all three *L*-edges, even if collection modes need to be changed from transmission or fluorescence geometries. It also shows its ability to quickly identify competing edges (the Yb *L*_II_- and *L*_I_-edges) informing the users to update scan ranges to improve throughput.

## 4. Conclusions

*CatMass* is an X-ray absorption sample mass calculator that is designed for complex sample inputs such as those used in the catalysis community. Features such as complex sample inputs, a competing edge finder and the ability to quickly iterate through calculations and save results for future reference expand on the capabilities of similar available software. With a Python-based development version as well as an executable, *CatMass* offers the ability for users to contribute to the project, allowing it to grow based on the needs of the community. Access to X-ray absorption sample mass calculators increases productivity by allowing experimentalists the ability to gauge the strength of an X-ray absorption event in a sample, and guiding them in detector selections and the overall complexity of the measurements prior to the experiment.

## Supplementary Material

Sections S1 to S3 including Figures S1 to S6. DOI: 10.1107/S160057752300615X/rv5174sup1.pdf

## Figures and Tables

**Table 1:** General mass ranges for XAS cells available for *ex situ* and *in situ* experiments

Sample	Edge	Cross-sectional area of sample holder (cm^2^)	TAL† at *E­*_0_ + 50 eV	Dilution ratio (mg_sample_:mg_diluent_)	Orientation to X-ray beam (°)	Usable *k*-range (Å^−1^)‡	Estimated edge step (based on preparation)	Measured edge step or fluorescence	Reference
7.5 µm-thick copper foil	Cu *K*	1.0	1.83	6.7:0	90	16+	1.58	2.0	This work
Yb­_2_O_3_	Yb *L*_III_	0.385	1.2	1.6:25.0§	90	15	0.61	0.48	This work
	Yb *L*_II_		1.2	1.6:25.0§	90	4	0.28	0.22	
	Yb *L*_I_		1.2	1.6:25.0§	45	16+	0.12	0.12	
10 wt% Co/TiO_2_	Co *K*	0.1	2.5	1.1:2.1	90	16+	0.32	¶	Van Ravenhorst *et al.* (2021 ▸)
0.05 wt% Pt/MgO	Pt *L*_III_	0.3	¶	∼50:0	45	16+	¶	Fluorescence measurement	Chen *et al.* (2022 ▸)
Re metal particles	Re *L*_III_	¶	¶	¶	90	16+	¶	¶	Qi *et al.* (2020 ▸)
0.5 wt% Pt/CeO	Pt *L*_III_	0.3	¶	∼50:0	45	16+	¶	¶	Resasco *et al.* (2020 ▸)

† Total absorption length.

‡ Usable *k*-range is defined as the *k*-space without competing metal edges.

§ The Yb_2_O_3_ *L*-edges were calculated such that a single sample preparation would allow measurement of all three edges.

¶ Not reported.
