# Supplementary material for: CatMass: software for calculating optimal sample masses for X-ray absorption spectroscopy experiments involving complex sample compositions
Source: J Synchrotron Radiat. 2023 Aug 18;30(Pt 5):1023–9. doi: 10.1107/S160057752300615X (PMC10481269; doi:10.1107/S160057752300615X)
Supplement: Supplementary file 1 [file s-30-01023-sup1.pdf]

JOURNAL OF SYNCHROTRON RADIATION

Volume 30 (2023)

Supporting information for article:

CatMass – Software for calculating optimal sample masses for X-ray absorption spectroscopy experiments involving complex sample compositions

Jorge E. Perez-Aguilar, Ash Caine, Simon R. Bare and Adam S. Hoffman

S1. Sample Building Examples

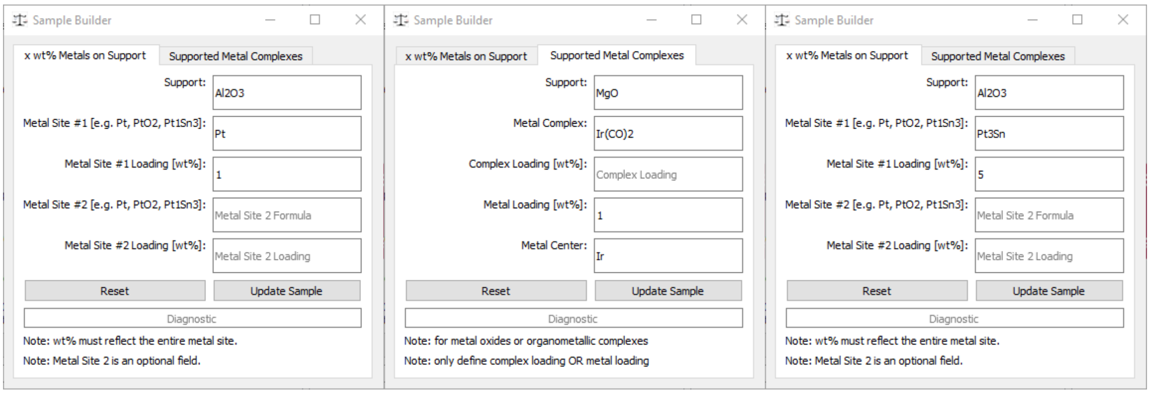

Figure S1 Examples of several complex catalysts when using the "Sample Builder" window; Left: 1 wt% Pt on Al_2O_3 (Aitbekova *et al.*, 2022); Middle: 1 wt% $Ir(CO)_2$ on MgO where the wt% is percent metal (Hoffman *et al.*, 2018); Right: 5 wt% Pt_3Sn nanoparticles on Al_2O_3 (Baraa Werghi *et al.*, 2023). Clicking "Update Sample" will convert these inputs into a stoichiometric composition and pass the values to the main window.

S2. Ytterbium oxide (Yb_2O_3) details

S2.1. Experimental methods

Extended X-ray absorption fine structure (EXAFS) measurements were conducted at beamline 10-2 at the Stanford Synchrotron Radiation Lightsource (SSRL). Beamline 10-2 has a 33-pole, 1.72-Tesla wiggler source and a double-crystal, liquid-nitrogen-cooled Si(111) monochromator. Step-scan spectra were collected in transmission mode with nitrogen-filled ion chambers. A Cu foil was scanned simultaneously with the sample for energy calibration.

S2.2. Choosing a reference foil for the measurement

When measuring a material that a reference foil would be difficult to obtain, e.g. Yb_2O_3, ***CatMass*** can be used to visualize where standard metal foils would appear in *k*-space for reference foil selection. Using the "Sample and Dilution Definition" panel, potential elements to be used as a reference can be input as "diluents". When the plot option is selected when calculating the results, the edges of the potential references are displayed in the *k*-space plot. In the example presented in Fig. S2, when scanning the Yb L_{III}-edge either the Cu or Zn reference foils could be used as a reference for internal energy calibration.

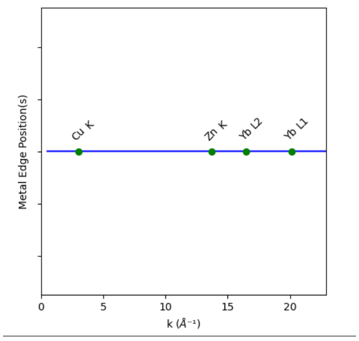

Figure S2 *K*-space of ytterbium L_{III} edge where the K edges of copper and zinc foils appear at k = 3.0 $Å^{-1}$ and k = 13.6 $Å^{-1}$, respectively. Data point density during the post-edge region near the metal foil edge would need to be modified to ensure a high enough resolution to use Cu or Zn as a reference for energy calibration.

S2.3. CatMass calculations

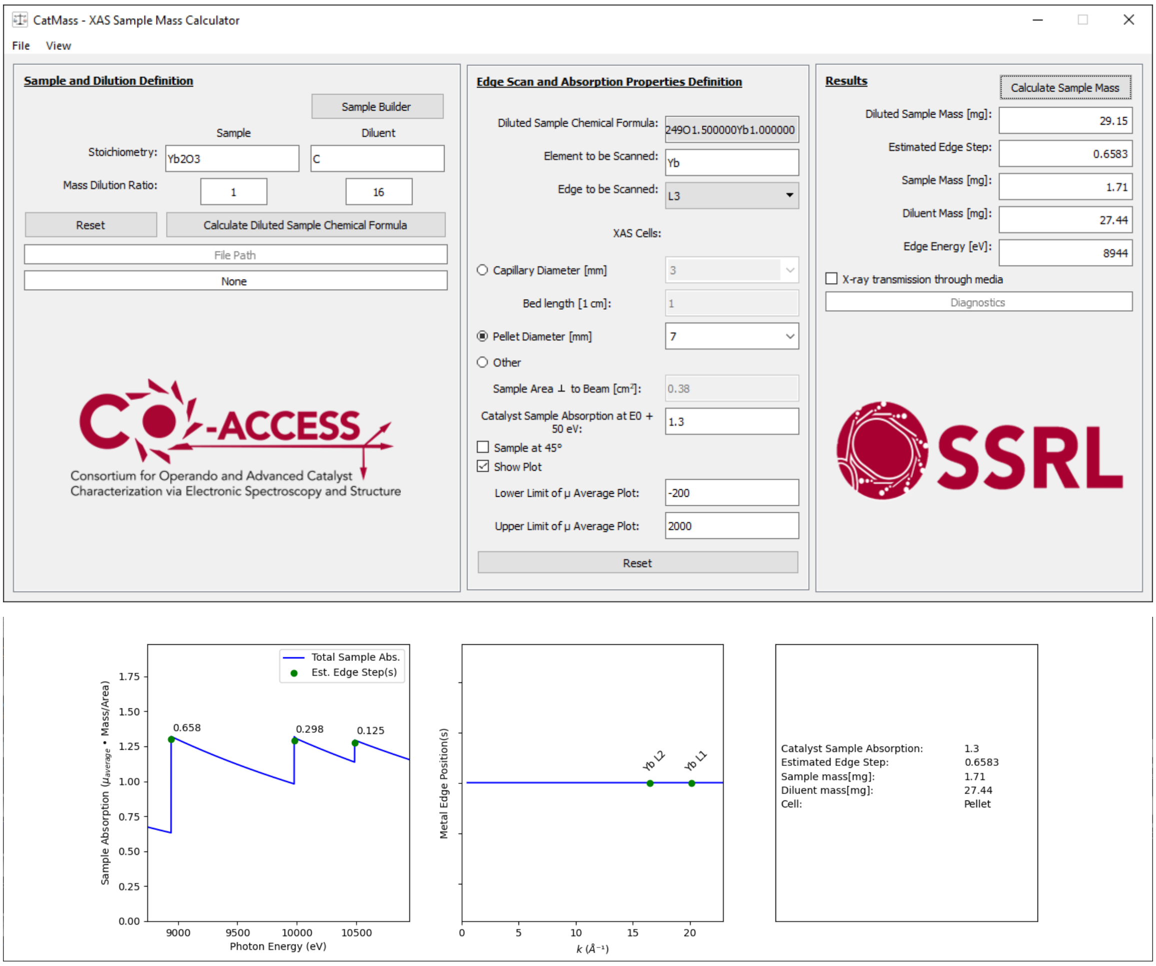

Figure S3 Example of a Yb_2O_3 pellet diluted with cellulose at a 1:16 ratio. Calculation is optimized for a pellet to be place 90º relative to beam path with a total mass of approx. 29 mg. The plotting limit is extended to display the other edges of interest (edges L_{II} and L_I of ytterbium as shown in the *k*-space plot). Transmission spectra should be recorded for L_{III} and L_{II} edges, and fluorescence for the L_I edge base on the total absorption values in the photon energy plot.

S3. Example calculation of alternative solutions when using CatMass

S3.1. Smear on tape

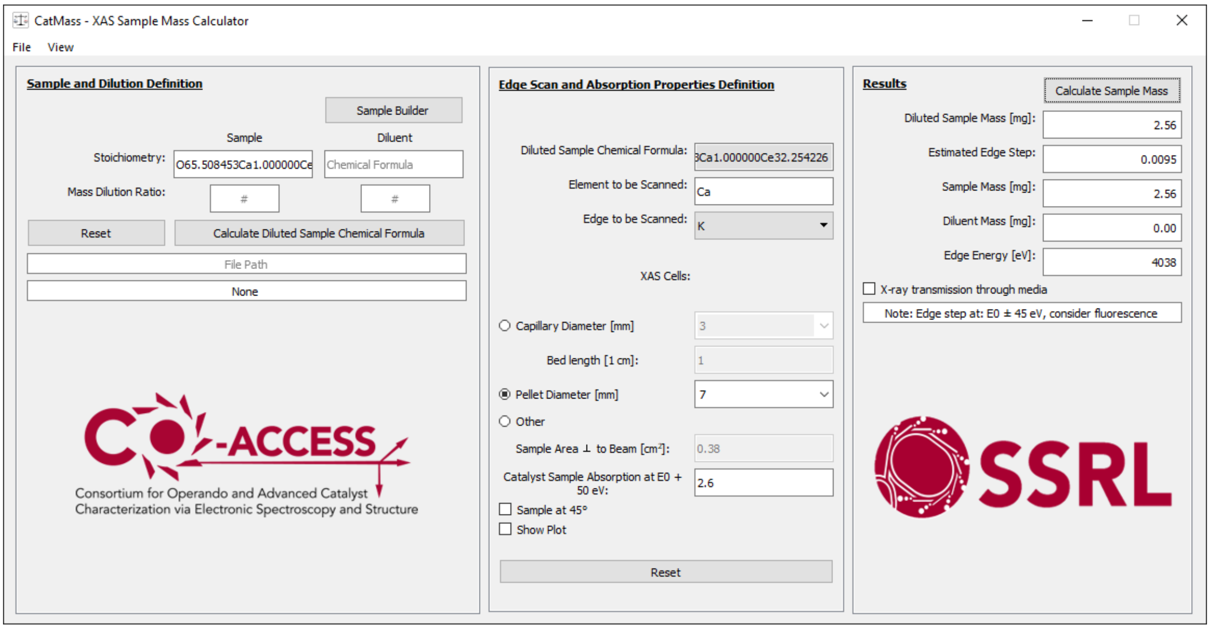

Figure S4 Example of 1wt%CaO/CeO$_2$. Calculation could not be optimized with a diluent to prepare enough sample material into a 7 mm pellet. Based on the sample mass value, smearing the sample would make the most physical sense to prepare inside a glovebox.

S3.2. Stacked pellets

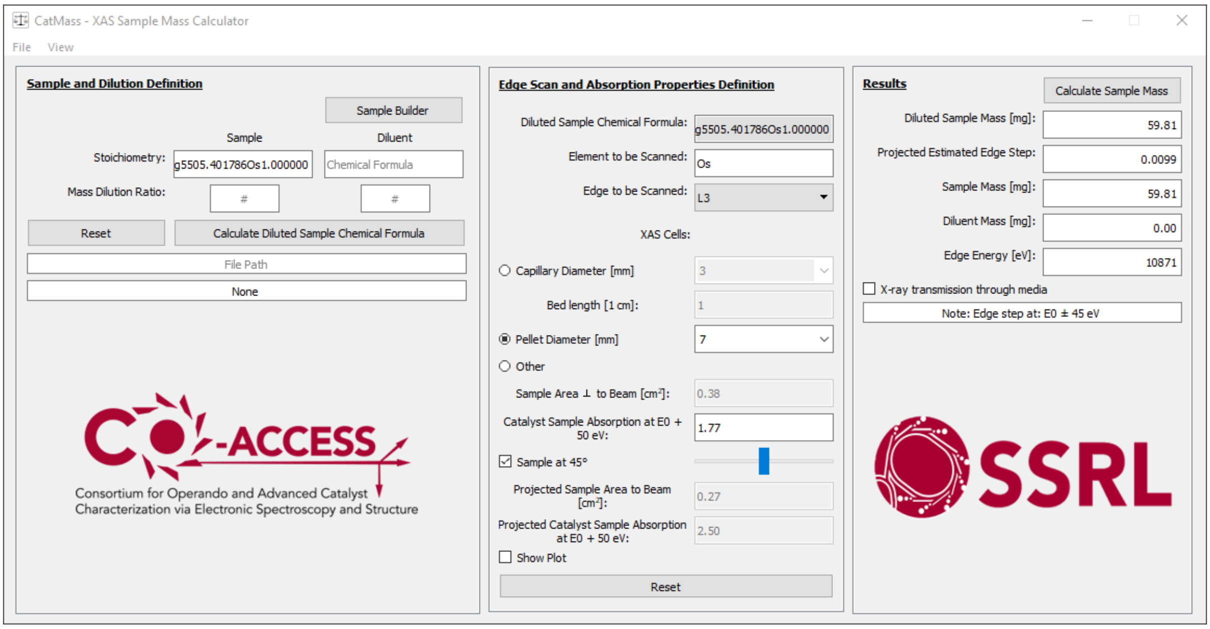

Figure S5 Example of 0.1wt%OsO$_2$/MgO. Calculation could not be optimized to satisfy the mass requirement range of one 7 mm pellet and keep a reasonable edge step for fluorescence. However, two pellets stacked on top of each other would add up to the estimated mass needed.

S3.3. Total absorption measurement

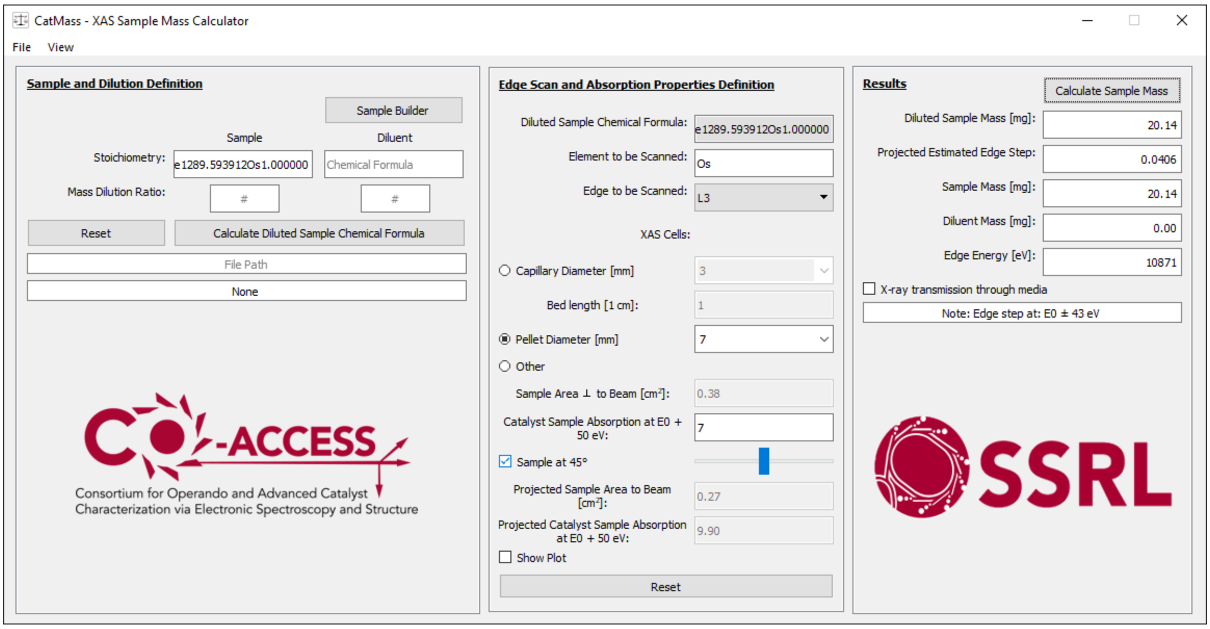

Figure S6 Example of 0.1wt%OsO_2/CeO_2. Calculation could not be optimized to satisfy the mass requirement range of one 7 mm pellet and keep a reasonable edge step for fluorescence. Increasing the sample absorption to meet the minimum requirement of packing the sample in a 7 mm pellet would be ideal. Smear on tape could yield measurable data, however the edge step is an order magnitude smaller and would require a high number of scans. Regardless, the use of a solid-state detector would be needed for the measurement and a simultaneous off axis reference foil measurement.

References

Aitbekova, A., Zhou, C., Stone, M. L., Lezama-Pacheco, J. S., Yang, A. C., Hoffman, A. S., Goodman, E. D., Huber, P., Stebbins, J. F., Bustillo, K. C., Ercius, P., Ciston, J., Bare, S. R., Plessow, P. N. & Cargnello, M. (2022). *Nat. Mater.* **21**, 1290–1297.

Baraa Werghi, Amani Ebrahim, Simon Bare & Matteo Cargnello (2023). *Small*, 2207956.

Hoffman, A. S., Debefve, L. M., Zhang, S., Perez-Aguilar, J. E., Conley, E. T., Justl, K. R., Arslan, I., Dixon, D. A. & Gates, B. C. (2018). *ACS Catal.* **8**, 3489–3498.
